# An unusual case of mesenteric fibromatosis

**DOI:** 10.1002/kjm2.12817

**Published:** 2024-03-01

**Authors:** Yu‐Cheng Chiang, Po‐Hsuan Wu

**Affiliations:** ^1^ Department of Clinical Education and Training Kaohsiung Medical University Hospital Kaohsiung Taiwan; ^2^ Graduate institute of medicine, College of Medicine Kaohsiung Medical University Kaohsiung Taiwan; ^3^ Department of General Surgery Kaohsiung Medical University Hospital Kaohsiung Taiwan

A 35‐year‐old male presented at our emergency department with a 2‐week history of abdominal fullness, pain, and vomiting. He had no underlying health conditions, previous surgeries, or familial cancer history. Physical examination and lab tests in the emergency room were unremarkable, revealing no palpable abdominal mass. However, an abdominal computed tomography (CT) scan showed a 7.5 × 5.2 cm mesenteric soft tissue mass, suggestive of a gastrointestinal stromal tumor (GIST) involving the small bowel, likely causing mechanical ileus (Figure 1A, arrow). During laparotomy, a 7.5 × 4.5 × 3.5 cm mass was found in the mesentery of the jejunum, attached to the jejunum and T‐colon. Resection of the mesenteric tumor (Figure 1B, arrowhead), along with a segment of the jejunum and a segment of the transverse colon, was successfully performed, followed by end‐to‐end anastomosis of the jejunum and T‐colon separately. Microscopic examination revealed spindle and stellate cells exhibiting vesicular nuclei with small nucleoli (Figure 1C). Immunohistochemistry showed that the tumor cells were positive for β‐catenin and exhibited focal expression of SMA, desmin, and H‐caldesmon, while being negative for S‐100, DOG‐1, and CD117. These characteristics are typical of intra‐abdominal mesenteric fibromatosis (MF) (Figure 1D).

**FIGURE 1:**
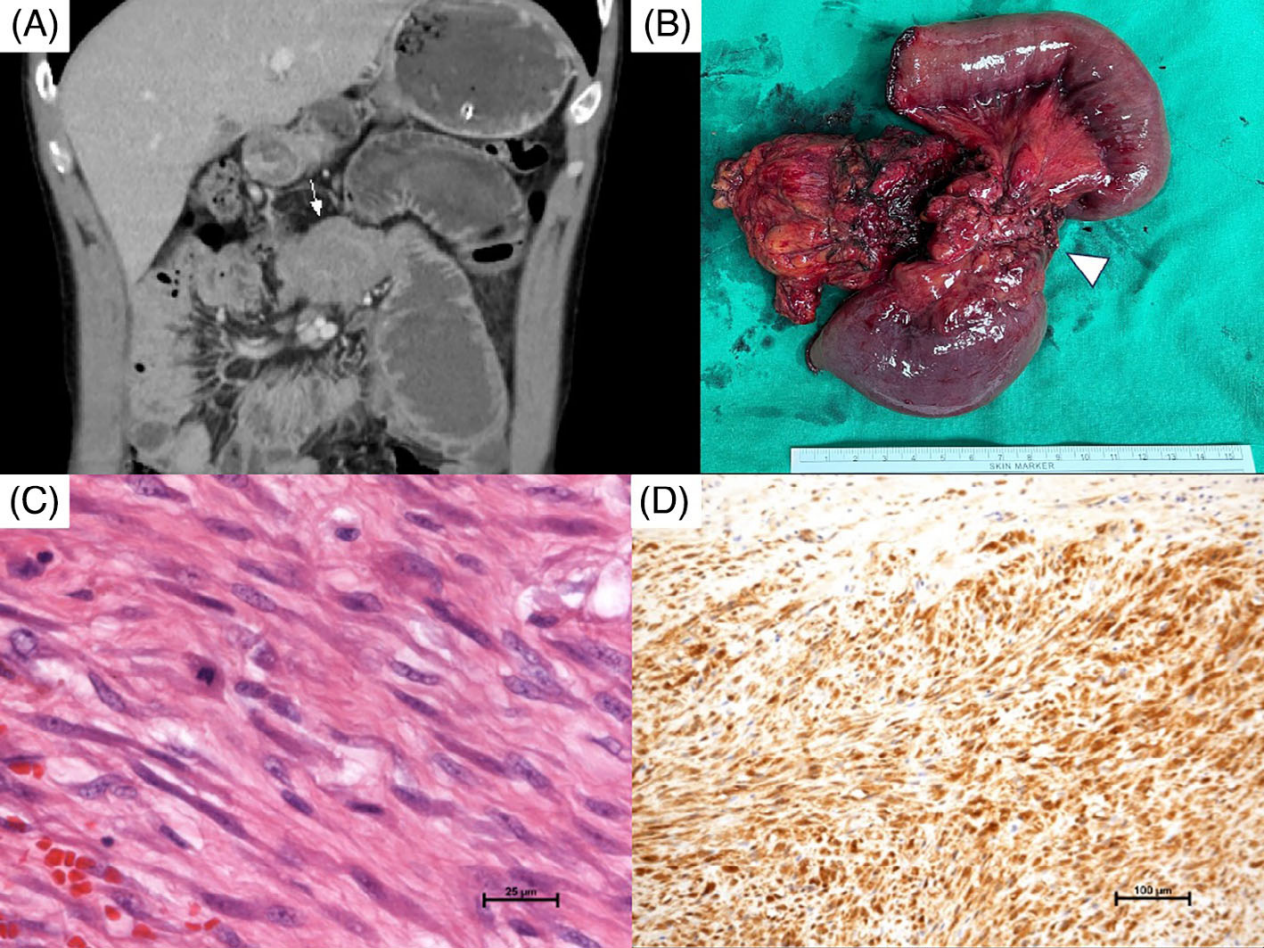
(A) Abdominal computed tomography revealed a 7.5 × 5.2 cm mesenteric soft tissue mass, suggestive of a gastrointestinal stromal tumor (GIST) involving the small bowel, likely causing mechanical ileus (white arrow). (B) Gross examination disclosed a 7.5 × 4.5 × 3.5 cm mass in the mesentery of the jejunum, attached to both the jejunum and the T‐colon (white arrowhead). (C) Microscopic examination revealed spindle and stellate cells exhibiting vesicular nuclei with small nucleoli. (D) Immunohistochemistry showed that the tumor cells were positive for β‐catenin and exhibited focal expression of SMA, desmin, and H‐caldesmon, while being negative for S‐100, DOG‐1, and CD117.

Desmoid‐type fibromatosis (DTF) is a rare tumor, constituting only 0.03% of all neoplasms,^1^, ^2^ predominantly affecting individuals in their 30s and 40s, with a female‐to‐male ratio of 2:1.^1^ Desmoid fibromatosis can develop in various body parts, typically involving the limbs, abdominal wall, and less frequently, the abdominal cavity, especially the mesentery. Patients with DTF may initially be asymptomatic, but as the tumor enlarges, it can press on nearby organs, leading to complications like intestinal obstruction, ischemia, and bleeding.^3^, ^4^ The etiology of fibromatosis is unclear, but potential contributing factors include trauma, estrogen levels, genetic predisposition (e.g., familial adenomatous polyposis [FAP]), and autoimmune disorders like Crohn's disease.^1^ About 30% of sporadic DTF cases follow trauma, indicating that abnormal cell proliferation in wound healing. The incidence of DTF in FAP patients is around 1000 times higher than in the general population,^4^ with 5%–15% of DTF cases occurring in FAP individuals.^2^ Additionally, β‐catenin mutations are identified in 71%–91% of spontaneous DTF cases.^2^ Accurate diagnosis of MF is critical, as misdiagnosis can result in inappropriate treatment. Grossly, MF appears as solid, grayish, uniform masses, differing from the softer, variable GISTs.^3^, ^5^ On CT, MF is typically well‐defined and homogeneous, contrasting with the heterogeneous, often necrotic GISTs. On magnetic resonance imaging (MRI), MF mostly appears hypointense on T2‐weighted images.^3^, ^5^ Diagnosis relies on microscopic analysis and immunostaining, with β‐catenin expression being a key identifier.^3^, ^5^ Treatment for MF is debated due to its unpredictable nature. Observation is preferred for slow‐growing or regressing tumors,^5^ while surgery is the primary treatment for progressive, operable MF.^2^ Achieving microscopically negative for residual tumor (R0 surgical margins) is crucial for reducing recurrence rates.^2^ Aggressive surgery can lead to complications like short bowel syndrome. Radiotherapy is controversial due to radiation enteritis risks. For microscopically positive residual tumor (R1 resection), postoperative recurrence, or unresectable tumors, systemic treatments like chemotherapy, anti‐estrogenic therapy, and molecular‐targeted therapy are options. Despite available treatments, recurrence rates are high (24%–77%), necessitating regular monitoring through CT or MRI scans.^3^ In conclusion, mesenteric DTF is uncommon, marked by slow yet locally invasive growth. While symptoms like ileus are infrequent, they can occur, as demonstrated in our case. Surgical removal is the preferred initial treatment, yet the high rate of recurrence continues to be challenging.

## CONFLICT OF INTEREST STATEMENT

All authors declare no conflict of interest.
